# Flora of Gorongosa National Park: a review of a modern African environment and its implications for baboon diet

**DOI:** 10.1007/s10329-026-01278-9

**Published:** 2026-07-09

**Authors:** Emanuela Rabajoli, Susana Carvalho

**Affiliations:** 1 Interdisciplinary Centre for Archaeology and the Evolution of Human Behaviour (ICArEHB), Universidade do Algarve, Faro, Portugal; 2 Centro de Investigação em Biodiversidade e Recursos Genéticos, CIBIO/InBIO, Universidade do Porto, Campus de Vairão, Vairão, Porto, Portugal; 3 Department of Science, Gorongosa National Park, Chitengo, Sofala, Mozambique; 4 School of Anthropology and Museum Ethnography, University of Oxford, Oxford, UK

**Keywords:** Chacma baboons, Dietary flexibility, Seasonality, Plant–animal interactions

## Abstract

What do we know about how the most abundant species of primates in Gorongosa National Park (Mozambique) exploit a highly seasonal and heterogeneous environment? This review examines current knowledge and key gaps in our understanding of Gorongosa’s vegetation in relation to the diet and feeding ecology of grey-footed chacma baboons (*Papio ursinus griseipes*). By synthesising existing literature of Gorongosa’s historical context, seasonality, landscape structure, and plant communities, this review provides environmental context for understanding baboon feeding ecology. Baboons appear to be among the most numerous mammals in Gorongosa National Park, inhabiting diverse landscapes and utilising the environment dynamically. Gorongosa National Park provides a useful contemporary system for investigating wild living primates, as it is a highly seasonal area with mosaic environments and a complex history, and it offers a valuable ecological analogue for early hominin environments. In this review, previous work on Gorongosa’s chacma baboon ecology and diet is synthesized, highlighting their pronounced dietary flexibility, foraging strategies, and extensive resource use. We also briefly consider their possible ecological role in seed dispersal, vegetation dynamics, and interactions with invasive plants, as these aspects are poorly documented in the park. Overall, this work organises existing data on baboon feeding ecology in Gorongosa, identifies key gaps, and outlines priorities for future integrative research, contributing to broader discussions of primate ecology, conservation, and human evolutionary models.

## Introduction

**Fig. 1:**
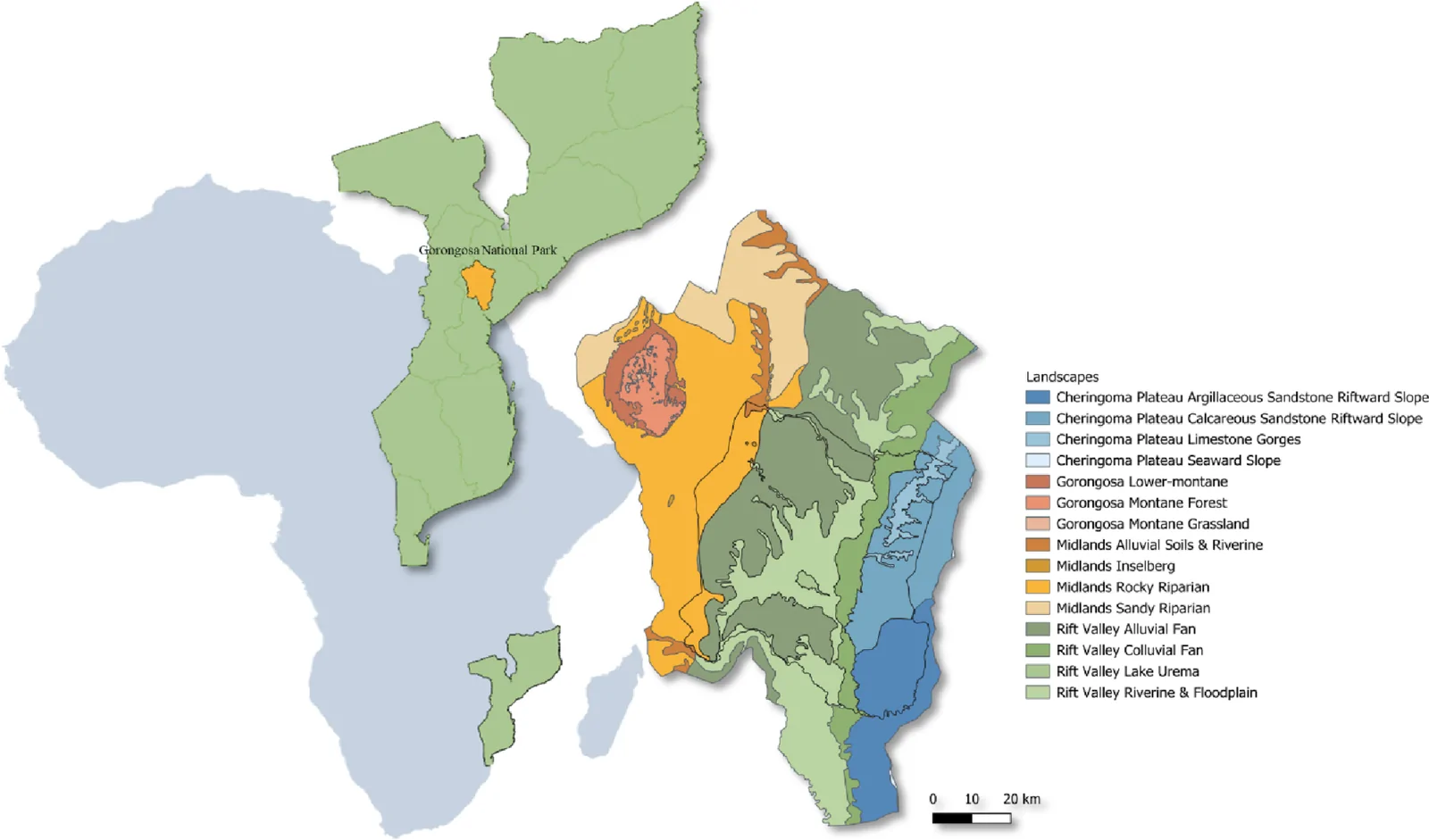
Location and landscapes of the Gorongosa National Park and Buffer Zone, Sofala province (Mozambique)

Gorongosa National Park (GNP) is a highly seasonal area located at the southern end of the East African Rift System in the Sofala Province of Mozambique (Fig. 1). It is one of the world’s great ecological hotspots, with notable biodiversity and heterogeneous habitats, including closed-canopy rainforest, savannah, montane grasslands, rivers, and caves (Tinley 1977; Stalmans and Beilfuss 2008). The area is characterised by strong gradients in elevation (from 15 to 1,900 m above sea level), rainfall, and hydrology. Precipitation increases with elevation, reaching 2,000 mm in Gorongosa Mountain. The Rift Valley, in the centre of the park, receives an average of 840 mm of rain annually (Tinley 1977; Stalmans and Beilfuss 2008). Numerous rivers and streams drain into the valley floor and Lake Urema, resulting in frequent and extended seasonal flooding across large areas of the Rift Valley (Daskin et al. 2016; Stalmans et al. 2019). Water availability and dynamics strongly influence vegetation distribution and structure, creating a mosaic of plant communities that reflect the region’s high ecological diversity (Stalmans and Beilfuss 2008). In addition, with its diverse landscapes, high seasonality, complex and rapidly changing environments, and historical complexity, Gorongosa National Park provides a modern ecological analogue to early hominin habitats (Bobe et al. 2020; Baehren and Carvalho 2022; Carvalho et al. 2025), resulting in an ideal site to study living primates.

Among the primates inhabiting Gorongosa National Park, grey-footed chacma baboons (*Papio ursinus griseipes*) are a highly adaptable, omnivorous species that occupies a broad range of landscapes across the park (Fleagle 2013; Stalmans and Peel 2024; Carvalho et al. 2025). Despite their prominence in the Gorongosa ecosystem, the current understanding of their diet remains fragmented across historical observations and more recent studies employing different methodologies. This review aims to synthesise existing information on Gorongosa’s flora and baboon feeding ecology. We begin by outlining the park’s history, seasonal dynamics, landscapes, and vegetation, before integrating available evidence on baboon diet and ecology to examine how resource availability and environmental heterogeneity shape feeding strategies.

### Historical context

Gorongosa National Park’s history is shaped by colonialism, conflicts, people and communities, wildlife loss and recovery, education, and an aspiring vision for the future. The history of GNP is not only one of rewilding and wildlife reintroductions, but also a commitment to benefitting local people through jobs in conservation, science, tourism, and education. In addition to running a Master’s Degree program in Conservation Biology, the park also facilitates educational projects that reconnect people with their heritage (https://gorongosa.org/*).*

Gorongosa was first established as a hunting reserve in 1920 by ‘The Mozambique Company’, a private business that managed the centre of Mozambique for the Portuguese government. In 1960, it was officially declared a national park, expanding from 1,000 to 5,300 square kilometres. Toward the end of that decade, the park’s ecologist Kenneth Tinley began systematic aerial wildlife surveys, leading the first comprehensive count in 1969. His team documented extraordinary numbers of large mammals, revealing one of Africa’s most abundant ecosystems at the time. His monumental PhD thesis, “Framework of the Gorongosa ecosystem”, remains the definitive ecological work on Gorongosa and is still used as a reference tool by the park management today (https://gorongosa.org/timeline/*)* (Tinley 1977).

In 1975, Mozambique obtained independence from Portugal, and in the following years (from 1977 to 1992), the Mozambican Civil War devastated the country. During and immediately after the conflict, Gorongosa National Park’s wildlife was heavily hunted for food and sale, including ivory used to finance the war. The first post-war aerial census in 1994 revealed a catastrophic decline of large herbivore populations, with an estimated 96% loss in biomass (Hatton et al. 2001; Stalmans et al. 2019). Many of Gorongosa’s iconic large grazers and predators had vanished.

In 2004, the Gorongosa Restoration Project, a US-based non-profit organisation, was established to restore the park and create a successful and now well-known wildlife restoration project (Pringle 2017). These recovery efforts have brought back species, rebuilt ecosystems, and engaged local communities in conservation, adopting 21st-century conservation models. In 2005, a 62 sq km fenced wildlife sanctuary was built in preparation for the large-scale wildlife restocking effort. From 2006, animals were introduced or relocated, starting with buffalo, wildebeest (2007), elephants and hippos (2008), eland (2013), wild dogs (2018), leopards (2020) and hyenas. The results of these efforts can be seen in the last aerial counts. The initial surveys conducted by Tinley from 1969 were carried out with a fixed-wing aircraft and after the year 2000, with a helicopter to be able to tally smaller species (Stalmans and Peel 2024). Since 1969, 18 wildlife counts have been carried out; the counts from 2014 to 2024 (Stalmans et al. 2014, 2018, 2022; Stalmans and Peel 2016, 2020, 2024) are inserted into an ACCESS database that holds more than 130,000 individual records (Stalmans and Peel 2024). In parallel with wildlife restoration, the project expanded its scientific capacity, launching long-term biodiversity surveys in 2013 and establishing a dedicated biodiversity laboratory to support ecological research and monitoring.

### Seasonality

**Fig. 2:**
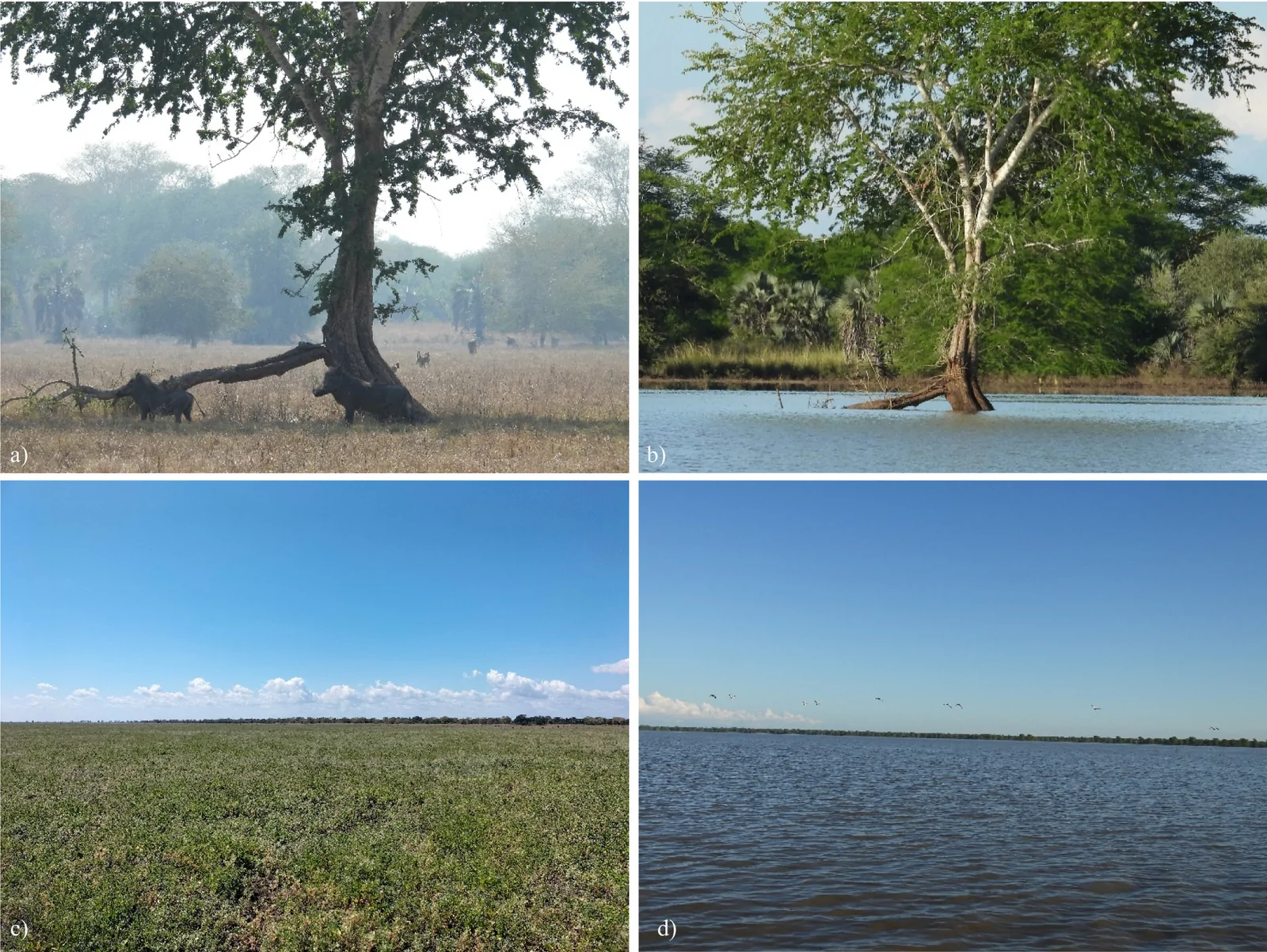
Seasonal variation in Gorongosa National Park illustrated through photographs of the floodplain. Picture (**a**) was taken on the 4^th^ of September 2025, picture (**b**) (representing the same tree) was taken on the 17^th^ of April 2026. Picture (**c**) was taken on the 18^th^ of August 2025, while picture (**d**) was taken on the 17^th^ of April 2026

The area is defined by strong seasonal variability, with a dry season lasting from April to October and a rainy season from November to March (Tinley 1977). Temperatures can exceed 40 °C in open areas during the dry season, and may drop to around 5 °C at night in some locations (Herrero et al. 2020). Gorongosa National Park can be flooded by up to 40% during the peak of the rainy season, significantly altering habitat availability and connectivity (Stalmans and Beilfuss 2008; Herrero et al. 2020; Beardmore-Herd et al. 2025). The most visible example of these variations is in the area around Lake Urema and the floodplain (Fig. 2). Seasonal rivers can fill up and eventually dry into small ponds or empty riverbeds.

Annual rainfall variability can be a key factor in habitat fluctuation. Herrero et al. (2020) identified seasonal variation in precipitation, and their results showed a decreasing trend from 2000 to 2016. The area can also be affected by major weather events, such as cyclones, severe floods, droughts, and wildfires. Fires are generally common in savanna and grassland habitats at the end of the dry season (Stalmans and Beilfuss 2008; Daskin et al. 2016). In 2019, Cyclone Idai hit central Mozambique, devastating and submerging human communities and significantly impacting Gorongosa’s wildlife (Walker et al. 2023; Beardmore-Herd et al. 2025).

These seasonal dynamics also influence the distribution and accessibility of resources across habitats. In the floodplain, inundation and subsequent water recession create shifting and dispersed resource patches, with different food types becoming available at different times of the year (Lewis-Bevan et al. 2025a). In contrast, woodland areas provide more stable conditions across seasons. The local population of baboons respond to this variability by adjusting their ranging behaviour and habitat use, shifting their movements and increasing terrestrial activity when access to food and water becomes more limited (Hammond et al. 2025; Lewis-Bevan et al. 2025a).

### Landscapes of the park

The park includes a wide range of vegetation types; different plant communities coexist within landscapes, and their combination typifies each distinct landscape. Tinley (1977) described 4 regions in GNP and the Buffer Zone around the park: Gorongosa Mountain Region, Midlands Region, Rift Valley Region, and Cheringoma Plateau Region. Inside those defined regions, Stalmans and Beilfuss (2008) identified 15 broad landscapes (Fig. 1; Table 1) (Tinley 1977; Stalmans and Beilfuss 2008). The objective of this work was to integrate vegetation and environmental factors to produce a landscape map of Gorongosa for research, monitoring, and land-use planning, as a detailed vegetation map was not feasible given the area’s large size and limited accessibility (Tinley 1977; Stalmans and Beilfuss 2008).

According to Stalmans and Beilfuss (2008), Gorongosa Lower Montane Grassland and Woodland Landscape contains a mosaic of grassland, shrub and woodland communities with scattered riverine elements and fragments of miombo woodland (e.g., *Brachystegia tamarindoides* and *B. spiciformis*), reflecting an upland savanna transition from surrounding miombo. Gorongosa Montane Forest Landscape is composed of sub-montane forest communities with canopy heights that decrease with elevation. This landscape shows fragmentation due to deforestation and cultivation. The Gorongosa Montane Grassland and Shrub-forest Landscape consists of large glades of montane grasslands and shrub-forest communities (such as Ericoid Scrub and *Erica hexandra*–*Rhytidosperma macowanii*) (Tinley 1977; Stalmans and Beilfuss 2008).

The Midlands Moist Miombo Landscape is considered the most transformed area by cultivation and is composed of moist *Brachystegia* (miombo) savanna woodland communities. Midlands Dry Miombo and Mixed Woodland Landscape is characterized by mixed savanna woodlands dominated by *Acacia–Combretum* types with limited miombo elements. Midlands Alluvial Landscape is located along larger rivers with a mix of open to closed woodlands and species associated with riverine forests and thickets. This landscape has also been heavily modified by cultivation. Midlands Inselberg Landscape vegetation consists of closed miombo woodlands with a more open canopy on rocky outcrops (Tinley 1977; Stalmans and Beilfuss 2008).

Rift Valley Alluvial Fan Landscape shows a complex mix of open to closed woodlands and dry forest/thicket communities adapted to alluvial fan soils; the most extensive community is *Acacia–Combretum* open to tall woodland. The Rift Valley Riverine and Floodplain Landscape is composed of a mix of mostly open plant communities, ranging from pure grasslands to sparse palm veld and open *Vachellia xanthophloea* and *Faidherbia albida* woodlands, and also includes some invasive species (*Mimosa pigra*). The Rift Valley Colluvial Fan Landscape is characterised by a mixture of open to closed woodland communities, and the Rift Valley Lake Urema Landscape is defined by Lake Urema’s water and dynamics, with some invasive water plants such as *Eichhornia crassipes* (water hyacinth) (Tinley 1977; Stalmans and Beilfuss 2008).

Cheringoma Plateau Seaward Slope Landscape consists of closed miombo woodlands with more open woodland and grassy dambo. Cheringoma Plateau Calcareous Sandstone Riftward Slopes Landscape has closed miombo woodlands with grassy dambo. Cheringoma Plateau Argillaceous Sandstone Riftward Slopes Landscape presents patches of open *Combretum* woodlands and closed *Brachystegia* woodlands. Cheringoma Plateau Limestone Gorge Landscape includes dry woodlands capped by *Androstachys johnstonii* (Tinley 1977; Stalmans and Beilfuss 2008).

Plant communities associated with each landscape and region are summarised in Table 1.

**Table 1:** Plant communities in landscapes of each region of Gorongosa National Park (adjusted from Stalmans and Beilfuss 2008)

Region	Landscape	Plant communities
Gorongosa Mountain Region	Gorongosa Lower Montane Grassland & Woodland	Grassland/secondary grassland with scattered trees; secondary woodland & forest; *Brachystegia tamarindoides* woodland; *Brachystegia spiciformis* woodland; tall miombo riverine forest
	Gorongosa Montane Forest	*Syzygium guineense* subsp. *afromontanum* montane forest; mixed sub-montane forest; medium altitude forest
	Gorongosa Montane Grassland & Shrub-forest	Montane grassland; submontane grassland; montane wetland (bogs, marshes, streams); wooded grassland on rock faces; ericoid scrub; *Erica hexandra – Rhytidosperma macowanii* open scrub; *Widdringtonia nodiflora* forest
Midlands Region	Midlands Moist Miombo	Short open to closed pan grassland; tall *Setaria* floodplain grassland; *Combretum* short open woodland in miombo; tall miombo riverine forest; tall closed miombo – *Acacia nigrescens* woodland; short hill-top miombo with *Sterculia quinqueloba*; Acacia–Combretum–Milletia short open woodland
	Midlands Dry Miombo & Mixed Woodland	Short open to closed pan grassland; *Combretum* short open woodland in miombo; tall miombo riverine forest
	Midlands Alluvial	Short open to closed pan grassland; tall *Setaria* floodplain grassland; riverine woodland and thicket communities
	Midlands Inselberg	Short hill-top miombo with *Sterculia quinqueloba*; miombo woodland on rocky outcrops
Rift Valley Region	Rift Valley Alluvial Fan	*Piliostigma thonningii – Borassus aethiopium* closed woodland/dry forest; *Dichrostachys cinerea* tall/high closed shrubland; *Dalbergia melanoxylon* low closed woodland; *Acacia xanthophloea* open to closed tall woodland; Acacia sparse to open woodland with saline grassland; Acacia–Combretum open to closed short to tall woodland; short to tall dry forest/thicket
	Rift Valley Riverine & Floodplain	Short open to closed pan grassland; tall *Echinochloa – Vetiveria* floodplain grassland; tall *Setaria* floodplain grassland; *Cynodon dactylon – Digitaria swazilandensis* low grassland; *Hyphaene*/*Borassus*/*Phoenix* open to closed tall palm veld; *Acacia xanthophloea* woodland; *Faidherbia albida* woodland; Acacia–Combretum woodland
	Rift Valley Colluvial Fan	Short open to closed pan grassland; short to tall dry forest and thicket
	Rift Valley Lake Urema	Tall *Echinochloa – Vetiveria* floodplain grassland; submerged and floating-leaved aquatic communities
Cheringoma Plateau Region	Cheringoma Plateau Seaward Slopes	Low dambo grassland; *Combretum* short open woodland in miombo; tall miombo riverine forest; tall closed humid miombo woodland
	Cheringoma Calcareous Sandstone Riftward Slopes	Low dambo grassland; *Combretum* short open woodland in miombo; tall miombo riverine forest; tall closed miombo – *Acacia nigrescens* woodland; tall closed humid miombo woodland
	Cheringoma Argillaceous Sandstone Riftward Slopes	Low dambo grassland; *Combretum* short open woodland in miombo; tall miombo riverine forest; tall closed humid miombo woodland
	Cheringoma Limestone Gorge	Tall to high limestone gorge forest; tall miombo riverine forest

### Vegetation

Mozambique has exceptional biological diversity; nevertheless, its flora has been generally understudied, with existing knowledge fragmented or limited. The lack of comprehensive documentation extends to both flora and fauna, reducing the understanding of the country’s biodiversity (Darbyshire et al. 2019; Malatesta et al. 2023). Gorongosa National Park is also subject to this limitation. While GNP’s landscapes are outlined and identified, data on plant communities remain limited. Existing information comes either from focused studies, such as the vegetation survey of tree cover of Mount Gorongosa, or from general lists of plants in the Sofala province or in Mozambique (Stalmans and Beilfuss 2008; Darbyshire et al. 2019; Odorico et al. 2022; Stalmans and Victor 2023; Malatesta et al. 2023). The information can be found in separate sources, such as literature, online databases, and herbarium collections (Burrows et al. 2018; Darbyshire et al. 2019; Odorico et al. 2022; Malatesta et al. 2023). Darbyshire et al. described the endemic vascular plants of Mozambique and their conservation status, resulting in a checklist of 271 endemic taxa and 387 near-endemic taxa (corresponding to 235 and 337 species, respectively). Here, “taxa” refers to taxonomic units including species, subspecies, and varieties. They also described the taxa distribution, identifying in Sofala province 47 strict endemics to Mozambique, 105 strict and near-endemics to Mozambique, 16 provincial endemics, and 21 strict or near-endemic restricted to a single province. This work identified Gorongosa as one of the areas of high national and global importance for its assemblages of endemic and range-restricted taxa (Darbyshire et al. 2019).

Odorico and colleagues (2022) updated a checklist of Mozambican plants. They combined information from all the different sources and verified the nomenclature. The identification gave 7,099 taxa, belonging to 226 families and 1,746 genera, divided into 6,804 angiosperms, 257 pteridophytes, and 38 gymnosperms. Additionally, the extinction risk of various taxa was included, resulting in 158 listed as Vulnerable, 119 as Endangered, and 24 as Critically Endangered. They identified 740 vascular plant taxa in the Gorongosa National Park area (Odorico et al. 2022).

### Flora and fauna interactions

Biotic interactions between fauna and flora are important drivers of vegetation dynamics in Gorongosa National Park. These interactions can affect plant communities in different ways, from shaping tree cover to influencing the spread of invasive species and creating localised habitat heterogeneity. The following examples illustrate how fauna can influence vegetation structure and highlight areas where evidence is still limited.

Resource availability, biotic interactions, and disturbance (including from human activities and herbivores) are the primary factors structuring plant communities (Ward et al. 2002; Sankaran et al. 2005, 2008). Among these, large mammal herbivores significantly affect woody cover: browsers and mixed feeders (elephant, kudu, impala, eland, nyala and bushbuck) tend to be negatively associated with tree cover in savanna ecosystems (Sankaran et al. 2008). Changes in herbivore populations can therefore lead to substantial shifts in vegetation structure. For example, Daskin et al. (2016) compared estimates of tree cover across Gorongosa National Park derived from satellite imagery collected in 1977 (pre-war) and 2012, showing a 34% increase in tree cover following the loss of large mammals (Daskin et al. 2016). While the effects of large herbivores on woody vegetation are well documented, the role of primates remains poorly understood. However, given that baboons are currently among the most abundant species in the park according to the last aerial count (Stalmans and Peel 2024), they may also contribute to vegetation dynamics. Primates are widely recognised as seed dispersers, but their influence on tree cover and vegetation structure has received comparatively little attention in GNP (Chapman and Russo 2005; Pamla et al. 2024).

Fire regimes also play a key role in shaping vegetation structure and influencing the habitat use of large mammals in Gorongosa. Recently burnt sites are nutrient-rich and characterized by regenerating vegetation, thereby influencing the distribution of plant resources and the movements of grass-dependent species (Massad et al. 2024). In their study of ecosystem-wide responses to fire, Massad et al. (2024) examined multiple taxonomic groups (mammals, birds, reptiles and amphibians), but habitat-use analysis primarily focused on large mammal herbivores. Waterbucks, impalas and warthogs showed strong use of recently burned areas, likely due to post-fire regrowth (Massad et al. 2024). Baboons, which also rely on grasses and herbaceous species, may likewise be influenced by this fire-driven resource availability.

Mammals can confer biotic resistance against the encroachment of plants, such as the invasive shrub *Mimosa pigra*, which has spread in the park after the conflict. This plant has been documented for decades, but it has increased following defaunation (Guyton et al. 2020). Results from Guyton et al. (2020) showed that *Mimosa* accounted for a large proportion of the diets of the dominant ruminant species in the GNP floodplain, supporting a role of herbivory in limiting its encroachment. There is direct documented evidence of baboons feeding on *Mimosa pigra* in GNP (Tinley 1977), indicating that primates also interact with this species. Its abundance and value as a protein resource may play an important role in their diet. More generally, primates may function as both seed dispersers and seed predators depending on plant species and context, highlighting flexibility in their role in plant–animal interactions (Lukitsch and Elliott 2012; Pamla et al. 2024). Correia and colleagues (2017) identified primates (baboons and vervet monkeys) as the main seed dispersers in Gorongosa National Park, based on seeds without predation signs recovered from faeces collected inside and outside the sanctuary area. However, their results did not include *Mimosa pigra* and did not assess seed predation or species-specific dispersal for this invasive plant (Correia et al. 2017).

**Fig. 3:**
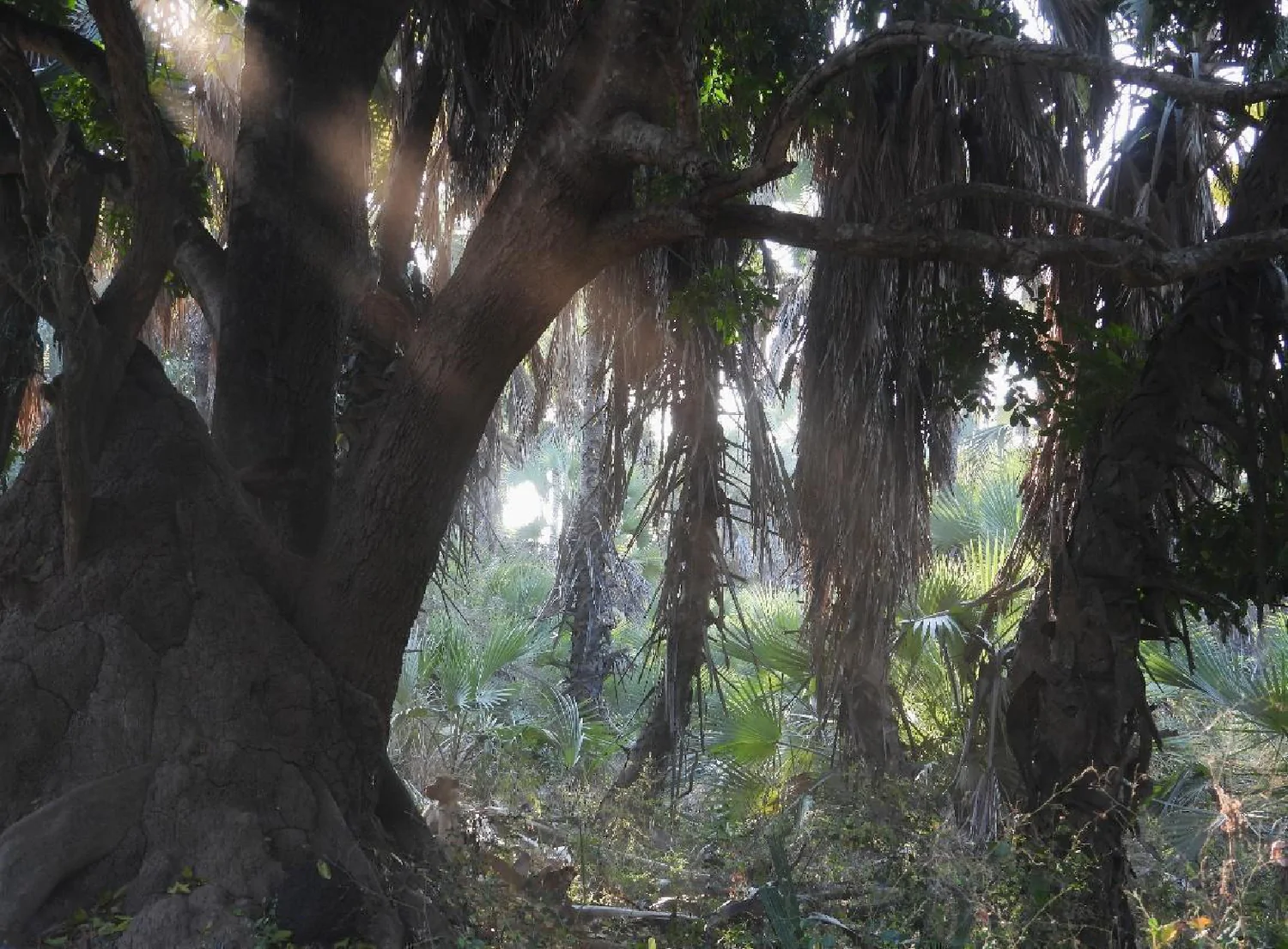
An adult baboon feeding at the base of a termite mound in Gorongosa National Park

Another example of the complex interaction between flora and fauna can be found in the ample termite mounds, created by fungus-farming termites of the genus *Macrotermes* (Macrotermitinae). By redistributing soil nutrients and retaining moisture, termites create localised, highly fertile zones around their nests (Daskin et al. 2016; Carvalho et al. 2025). These nutrient-rich patches are composed of distinctive plant assemblages that create elevated ecological refuges during floods or fires (Carvalho et al. 2025). These sites are preferentially used by herbivores (bushbuck, nyala, and kudu) and primates, including baboons and vervets (Fig. 3) (Tinley 1977; Daskin et al. 2016; Carvalho et al. 2025).

Overall, while the role of large herbivores in shaping vegetation structure is relatively well understood, the specific contribution of primates (baboons included) has limited documentation in GNP. This highlights an important gap in understanding plant-animal interactions within this ecosystem.

### Baboons in Gorongosa National Park

**Fig. 4:**
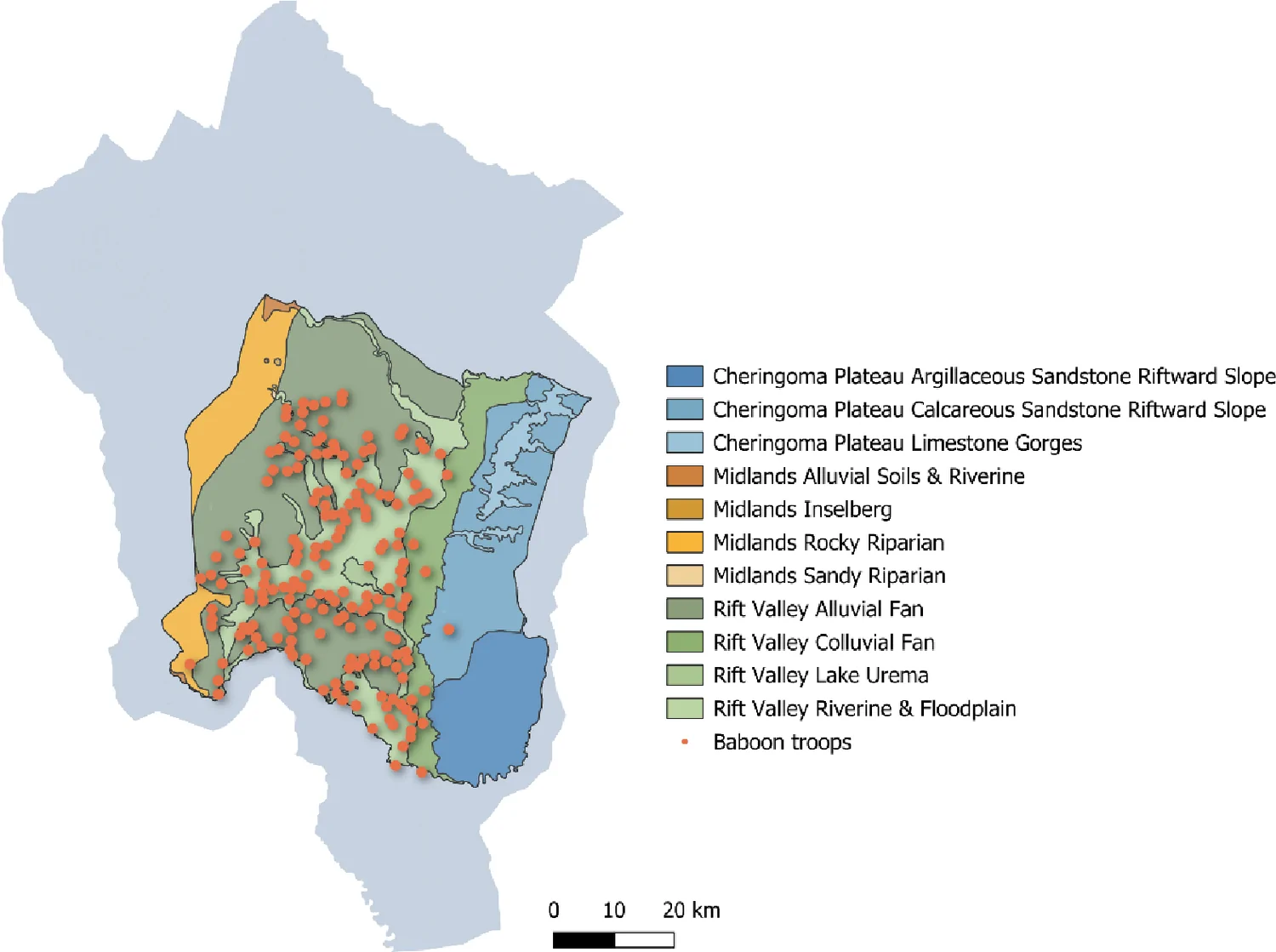
Baboon troops recorded in the last aerial count in Gorongosa National Park (Stalmans and Peel 2024)

Gorongosa currently is home to five primate species: baboons (*Papio ursinus griseipes*), vervet monkeys (*Chlorocebus pygerythrus pygerythrus*), samango (*Cercopithecus mitis erythrarchus*), large-eared greater galago (*Otolemur crassicaudatus*) and Mozambique dwarf galago (*Paragalago granti*) (Carvalho et al. 2025). Baboons in GNP are identified as a distinct subspecies of grey-footed chacma, reflecting a complex evolutionary history (Martinez et al. 2019; Ferreira Da Silva et al. 2025; Caldon et al. 2025). This species inhabits all the park landscapes successfully (Fig. 4), with a total of 229 baboon troops recorded in the last aerial count (Stalmans and Peel 2024). Baboon density went from < 0.05 to 0.15 troops/km^2^, based on observations from 1994 to 2018 (Stalmans et al. 2019).

**Fig. 5:**
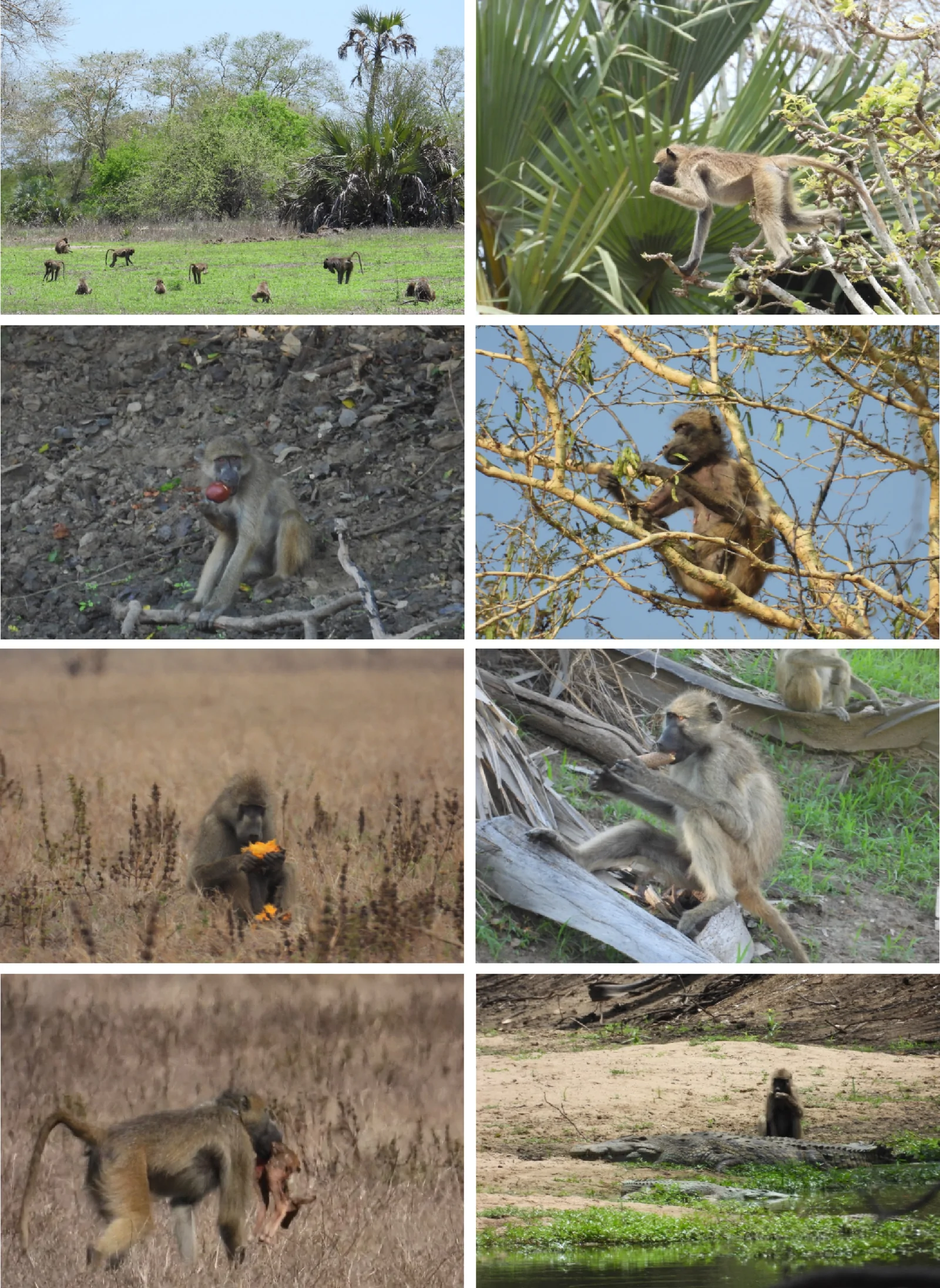
Examples of baboons feeding in Gorongosa National Park. **a** A group of baboons foraging in the floodplain. **b** A juvenile male feeding on *Kigelia africana* leaves. **c** One baboon eating a *Hyphaene coriacea* fruit. **d** An old female eating *Vachellia xanthophloea* seeds in the pods. **e** An adult male eating the *Borassus aethiopum* fruit. **f** An adult individual feeding on *Borassus aethiopum* inflorescence. **g** An adult male baboon hunting an infant reedbuck. **h** A pregnant female feeding close to two crocodiles

Previous studies on Gorongosa’s population of baboons documented pronounced behavioural and ecological flexibility. This includes the ability to adapt to extreme events (Beardmore-Herd et al. 2025), respond to strong seasonal variation in their home ranges, and display variation in movement patterns, including sleeping site selection and spatial memory of routes and resource patches (Lewis-Bevan et al. 2025a, b; Lewis‐Bevan et al. 2025). In addition, males show distinctive greeting behaviours (Baehren and Carvalho 2022; Muschinski 2025). This flexibility is particularly relevant in the context of Gorongosa’s ecological history. The severe biodiversity loss following the Mozambican Civil War (1977–1992) led to the consequential lack of habitual baboon predators (Hatton et al. 2001; Bouley et al. 2018; Stalmans et al. 2019; Hammond et al. 2022). This ecological context can possibly be relevant for interpreting patterns of baboon behaviour and ecology in the Gorongosa ecosystem (Fig. 5f).

### Baboons feeding ecology

Employing a selective, seasonal feeding strategy is an adaptive response to fluctuating resource availability and helps individuals maintain adequate energy intake across their lives (Alberts et al. 2005). Chacma baboons (*Papio ursinus*) are eclectic feeders; their diets consist of fruits, flowers, roots, tubers, seeds, gums and leaves (Fig. 5) (Hall and De Vore 1965; Fleagle 2013; Pebsworth 2020; Kennedy Overton et al. 2024). Occasionally, individuals can be opportunistic faunivores that hunt and eat small vertebrates, including mammals, birds, fish, and reptiles (Fig. 5g) (Pebsworth 2020; Hammond et al. 2022; Lambert et al. 2024). The composition of their diet and the feeding strategies differ between groups and individuals, influenced by local habitats, seasonal fluctuations in resources and individual preferences (Altmann 1998; Alberts et al. 2005).

While various aspects of Gorongosa baboons’ behavioural ecology (Hammond et al. 2025; Lewis-Bevan et al. 2025a, b, c; Farassi et al. 2025) and genomics (Martinez et al. 2019; Ferreira Da Silva et al. 2025; Caldon et al. 2025) are well studied, their feeding ecology remains underrepresented in the current literature. The first description of plants and plant parts consumed by baboons in Gorongosa National Park was by Tinley, who compiled a list of dietary items from 1968 to 1973, based on direct observation of feeding and dung. His work not only described the plants baboons rely on and how they exploit arboreal and ground-level foods, but also made evident their high flexibility and opportunistic diet, as well as the changes with season, habitat, and resource availability (Table 2) (Tinley 1977).

**Table 2:** List of plants and plant parts eaten by baboons, adapted from Tinley (1977)

Plant type	Wet Season	Plant part	Dry Season	Plant part
Grasses	*Echinochloa sp. nr. haploclada* *Echinochloa stagnina* *Eriochloa stapfiana* *Panicum coloratum* *Panicum maximum* *Paspalidium obtusifolium* *Paspalum scrobiculatum* *Urochloa mosambicensis* *Vossia cuspidata*	Fruits and seeds Fruits and seeds Fruits and seeds Fruits and seeds Fruits and seeds Clums Fruits and seeds Fruits and seeds Clums, roots and tubers	*Vetiveria nigritana*	Roots and tubers
Sadges	*Cyperus esculentus*	Roots and tubers		
Forbes	*Abutilon* spp. *Ludwigia stolonifera*	Flower	*Eichhornia crassipes* *Ludwigia stolonifera* *Pistia stratioites*	Roots and tubers Roots and tubers
Woody plants	*Acacia robusta* *Acacia sieberana* *Acacia xanthophloea* *Annona senegalensis* *Artabotrys monteiroae* *Berchemia discolor* *Borassus aethiopum* *Capparis erythrocarpos* *Cleistochlamys kirkii* *Diospyros mespiliformis* *Diospyros usambarensis* *Drypetes mossambicensis* *Ehretia amoena* *Ficus sycamorus* *Kigelia africana* *Manilkara mochisa* *Mimosa pigra* *Strychnos potatorum* *Thilachium africanum* *Vangueria infausta* *Xeroderris stuhlmannii* *Ximenia americana* *Xylotheca tettensis* *Ziziphus mucronata* *Ziziphus pubescens*	Gums, fruits and seeds Gums, fruits and seeds Gums Fruits and seeds Fruits and seeds Fruits and seeds Fruits and seeds Fruits and seeds Fruits and seeds Fruits and seeds Fruits and seeds Fruits and seeds Fruits and seeds Fruits and seeds Fruits and seeds Fruits and seeds Fruits and seeds Fruits and seeds Fruits and seeds Fruits and seeds Fruits and seeds Fruits and seeds Fruits and seeds Fruits and seeds Fruits and seeds	*Acacia albida* *Acacia robusta* *Acacia sieberana* *Albizzia harveyi* *Boscia salicifolia* *Brachystegia glaucescens* *Capparis erythrocarpos* *Diospyros mespiliformis* *Ficus sycamorus* *Ficus zambesiaca* *Friesodielsia obovata* *Hyphaene benguellensis* *Kigelia africana* *Mimosa pigra* *Mimusops fruticose* *Piliostigma thonningii* *Salvadora persica* *Sterculia appendiculata* *Tamarindus indica* *Thilachium africanum* *Trichilia capitata* *Xanthocercis zambesiaca* *Ximenia americana* *Ziziphus mucronata*	Fruits and seeds Fruits and seeds Fruits and seeds Fruits and seeds Fruits and seeds Fruits and seeds Fruits and seeds Fruits and seeds Fruits and seeds Fruits and seeds Fruits and seeds Fruits and seeds Fruits and seeds Fruits and seeds Fruits and seeds Fruits and seeds Fruits and seeds Fruits and seeds Fruits and seeds Fruits and seeds Fruits and seeds Fruits and seeds Fruits and seeds Fruits and seeds

A number of the plant species documented by Tinley (1977) in the diet of GNP baboons also featured in Sitoe & Van Wyk’s (2024) assessment of medicinal plant species and their uses in Mozambique (Tinley 1977; Sitoe and Van Wyk 2024). A representation of this overlap and the associated medicinal uses of the plant species consumed by baboons in GNP is summarised in Table 3, with the plant species reported by Tinley (1977) aligned with their corresponding nomenclature as used by Sitoe and Van Wyk (2024). However, it has not yet been investigated whether baboons in GNP select these species for their pharmacological properties. Evidence from South Africa suggests that such behaviour is possible: a year-long study on a troop of chacma baboons found that they consumed 68 plant species, of which 38 were reported as medicinal plants in the literature. For 28 of these, baboons ate specific parts with associated documented pharmacological properties (Pebsworth 2020). Considering that Tinley’s observations were not collected from habituated troops and were limited to what was easily observed, a deep investigation with habituated individuals would provide a clearer understanding of plant selection and its potential pharmacological basis.

**Table 3:** List of plants eaten by GNP baboons reported by Tinley (1977), corresponding species name and the medicinal use adjusted from Sitoe & Van Wyk (2024)

Species reported by Tinley (1977)	Corresponding species by Sitoe and Van Wyk (2024)	Medicinal use from Sitoe and Van Wyk (2024).	
*Acacia albida*	*Faidherbia albida*	Bloody vomit, cough, skin infection	
*Acacia xanthophloea*	*Vachellia xanthophloea*	Emetic, prophylactic	
*Albizzia harveyi*	*Albizia harveyi*	Diarrhoea, intestinal trouble	
*Annona senegalensis*	*Annona senegalensis*	Sexual complaints, stitches, sedative, blennorrhagia, dysenteries, throat diseases, vertebral column diseases, menstrual pains, epilepsy, wounds, wounds of the uterus, hydrocele, sexual potential stimulant, conjunctivitis, pharyngitis, spinal marrow disease, stomach-ache, intestinal wounds, cough, tuberculosis, fever, asthma, diarrhoea, respiratory, anthelmintic, venereal diseases, gastrointestinal disorders, ophthalmic, snakebites, astringent and tonic, abortifacient, inflammations, colics	
*Artabotrys monteiroae*	*Artabotrys monteiroae*	Unspecified	
*Berchemia discolor*	*Berchemia discolor*	Bloody vomit, cough	
*Borassus aethiopum*	*Borassus aethiopum*	Anti-phlegmatic, leprosy, aphrodisiac, tonic, laxative, diuretic	
*Boscia salicifolia*	*Boscia salicifolia*	Chills, pain in the eyes, oedema of the limbs, vomiting with diarrhoea	
*Capparis erythrocarpos*	*Capparis erythrocarpos*	Bronchitis, bronchitis and tuberculosis, headache, toothache, tuberculosis, stitches	
*Cleistochlamys kirkii*	*Cleistochlamys kirkii*	Haemorrhoids, rheumatism, tuberculosis, stomach-ache, vomit, muscular pains, venereal diseases, hernia, general weakness	
*Diospyros mespiliformis*	*Diospyros mespiliformis*	Propitiatory, dysentery, leprosy, relief for skin eruptions	
*Ehretia amoena*	*Ehretia amoena*	Diarrhoea, dysentery, stomach-ache, venereal diseases, malaria, muscle pain, vomiting	
*Eichhornia crassipes*	*Pontederia crassipes*	Female sterility	
*Ficus sycamorus*	*Ficus sycomorus*	Ringworm, toothache, cough, throat pain, skin inflammation, diarrhoea, chest conditions	
*Kigelia africana*	*Kigelia africana*	Fever, dysenteries, wounds, cauterization of the navel, diarrhoea, anal wounds, disinfectant, stomach-ache, toothache, syphilis, rheumatism, ulcers, purgative, gonorrhoea	
*Manilkara mochisa*	*Manilkara mochisa*	Toothache, ear treatment, epilepsy, worms, colics	
*Mimosa pigra*	*Mimosa pigra*	Diarrhoea, gonorrhoea, leprosy, emetic	
*Panicum maximum*	*Megathyrsus maximus*	Headache, febrifuge	
*Piliostigma thonningii*	*Piliostigma thonningii*	Bloody vomit, bilharzia, fever, backache, febrifuge, colds, cough, toothache, diarrhoea, dysentery, malaria, muscle pain, convulsions, ringworms	
*Pistia stratioites*	*Pistia stratioites*	Pertussis, ulcers, rheumatism, fever	
*Salvadora persica*	*Salvadora persica*	Respiratory system	
*Sterculia appendiculata*	*Sterculia appendiculata*	Unspecified	
*Tamarindus indica*	*Tamarindus indica*	Stomach-ache, tuberculosis, wounds, ulcers, abscesses, purgative, diaphoretic, emollient, liver complaints	
*Thilachium africanum*	*Thilachium africanum*	Asthma, rheumatism, oedema, fontanelle (clasp, vomiting with diarrhoea, skin injuries, digestive, muscular-skeletal, respiratory system	
*Vangueria infausta*	*Vangueria infausta*	Stomach-ache, diarrhoea, febrifuge (MK, oral hygiene, cough, induce/speed delivery, skin blisters, menstrual pain, toothache, epilepsy	
*Ximenia americana*	*Ximenia americana*	Anti-abortifacients, HIV/AIDS, menstrual cycle, stabbing heart, stomach-ache, women fertility, wounds	
*Xylotheca tettensis*	*Xylotheca tettensis*	Unspecified	
*Ziziphus mucronata*	*Ziziphus mucronata*	Roundworms, stomach-ache, constipation, muscular pains, diarrhoea, sexual complaints, female sterility	

In the years following Tinley’s study, Gorongosa baboons’ diet was not investigated until the work of Pansu et al. (2019). They used faecal analysis (specifically DNA metabarcoding) to quantify diet composition of the 13 most abundant large-mammal species in GNP and baboons: 8 grazers (waterbuck, reedbuck, warthog, oribi, sable, buffalo, hartebeest, wildebeest), 2 browsers (bushbuck and kudu), 3 mixed-feeders (impala, nyala, elephant), and 1 omnivore (baboon) (Tinley 1977; Pansu et al. 2019). Baboons are usually not considered large mammalian herbivores, but their importance in plant-animal interactions and abundance justified their inclusion in this research. The study provided a baseline description of the plant component of baboons’ diet in Gorongosa National Park, identifying the main plant families consumed: Fabaceae (28%), Malvaceae (24%), Moraceae (13%), and Arecaceae (12%) (Correia et al. 2017; Pansu et al. 2019).

Another milestone in the study of grey-footed chacma baboons’ diet, which actually focused on how baboons interact with vegetation, was Biro and colleagues’ (2025) study. The authors observed differences in bark-stripping of *Vachellia robusta* (formerly *Acacia robusta*) trees across troops using camera-trap videos and surveys. The results showed regional (and longitudinal) differences in the presence of this behaviour, suggestive of possible social transmission; in some areas, the target trees and baboon troops were documented, but bark stripping was not recorded (Biro et al. 2025).

Despite the contribution of Tinley (1977) and all the authors presented here, there is currently no comprehensive and updated list of items eaten by baboons in Gorongosa National Park. These studies provided important insight, with different methodological approaches, which limited direct comparison across datasets. Available information remains fragmented, representing a key gap in our understanding of baboons’ feeding ecology in Gorongosa.

### Evolutionary perspective

Similar to baboons, early hominins likely adjusted foraging strategies to cope with environmental stresses. Behavioural adaptations to temperature, scarce water, seasonal food availability, and predators were, and still are, vital for survival in open environments (King 2024). Cercopithecids (Papionini in particular) are very commonly found at hominin fossil sites and were the first primates studied as models for various aspects of human evolution, as they share physical (highly encephalized), behavioural, and ecological traits with early hominins (DeVore and Washburn 1963; Elton 2006; Dunbar 2009). They can be used with a comparative approach to explain the possible course of evolution, as through much of our evolutionary history, hominins and baboons occupied a similar dietary niche, with similar sociality and high terrestriality (DeVore and Washburn 1963; Dunbar 1983; Swedell and Plummer 2019; King 2022). This overlap extended to the exploitation of similar plants and plant parts (leaves, fruit, underground storage organs, seeds/pods). Peters and colleagues (1981) identified 6 botanical genera consumed by baboons, chimpanzees, and/or humans from the paleobotanical record and likely part of early hominins’ dietary niche: *Acacia*, *Albizia*, *Cordia*, *Diospyros*, *Ficus*, and *Ziziphus* (Peters et al. 1981).

Similarities in baboon and macaque societies support the idea that human sociality had been shaped by some of the same ecological pressures that influence the behaviours of extant terrestrial primates (Strier 2017). For example, dwelling on the ground can increase the risk of attacks from predators compared to being arboreal. This may explain why baboons live in large, coordinated troops (Strier 2017). Examining extant cercopithecid feeding ecology and sociality will help us understand the evolution of analogous adaptations of our ancestors when they shifted to more terrestrial life, increased group size, and broadened their diets, allowing them to occupy various world habitats successfully (Jolly 2001; Elton 2006; Macho 2014).

Paleoenvironmental studies of fossil wood documented, for the first time, Miocene coastal woodland and forest ecosystems in the southern East African Rift. These findings indicate that the GNP paleoenvironment included a mosaic of riverine forest/woodland and estuarine habitats along the coastal margin. The vegetation was dominated by C₃ woody plants, with fossil wood assemblages largely composed of *Entandrophragmoxylon* (African mahogany), followed by *Hyphaene* palms, *Terminalioxylon*, *Ziziphus*, and *Zanha* (Habermann et al. 2019; Bobe et al. 2023). In the Cheringoma area, wood similar to extant *Hyphaene* spp. (*Palmoxylon dutoitii*) was identified, as well as Anacardiaceae similar to the extant *Sorindeia madagascariensis* (*Sorindeioxylon gorongosense*) and Combretaceae (*Terminalioxylon mozambicens*e). The presence of this wood suggests an environment characterised by elevated groundwater levels, a warm frost-free environment, and moderate to high rainfall (Bamford and Pickford 2021). Currently, there is no evidence of a primate fossil record in GNP, but the discovery of a coastal forest landscape has raised the possibility that it may have been a key habitat for critical periods of primate evolution (Habermann et al. 2019; Bobe et al. 2023).

## Conclusions and future directions

In conclusion, Gorongosa National Park provides an exceptional setting for studying the ecology of living primates, the evolution of behaviour, and diet. Its unique mosaic of habitats, shaped by seasonal dynamics, historical disturbances, diverse landscapes, rich endemic flora, and abundant primate populations, offers valuable insights into feeding ecology, behavioural flexibility, and ecosystem functioning.

The studies presented here establish a baseline for ongoing and future research on the diets of baboons and other primates in Gorongosa. Future work would benefit from integrating different methodologies, specifically, direct observation with remote sensing techniques. The combination of traditional techniques used in primate diet research (faecal analysis, camera trap videos, radiocollar data, and phenology transects) is essential for achieving a more comprehensive understanding of baboon behavioural ecology, including dietary components beyond vascular plants. Especially considering that, after Tinley’s work (1977), there are no species-level studies or direct observational studies, and Gorongosa National Park underwent significant changes after that period.

In this context, the Paleo-Primate Project Gorongosa initiated long-term primatological field research in the park in 2016, providing a critical framework for exploring the evolutionary history of the Rift Valley ecosystem. The work focuses on multiple aspects of baboon ecology, sociality, evolution and genetics, aiming to address the current knowledge gaps by integrating different techniques and approaches. Future studies will benefit from these long-term efforts, particularly in the context of rapid climate change and the high year-to-year variability in GNP’s environmental dynamics. In this framework, highly flexible and adaptable species such as baboons represent valuable models for detecting and interpreting ecological and behavioural shifts over time. Existing evidence indicates that the ecological success of baboons in Gorongosa National Park is driven by their capacity to integrate spatially patchy and temporally fluctuating resources across a highly heterogeneous landscape.

## Data Availability

Not applicable.
